# An Approach for Gummy Smile Treatment Using Botulinum Toxin A: A Narrative Review of the Literature

**DOI:** 10.7759/cureus.34032

**Published:** 2023-01-21

**Authors:** Bader Fatani

**Affiliations:** 1 College of Dentistry, King Saud University, Riyadh, SAU

**Keywords:** treatment, gummy smile, gingiva, botulinum toxic a, botox

## Abstract

Excessive gingival exposure (gummy smile) is a non-aesthetic condition characterized by excessive exposure of the gingiva during smiling. The most common cause of gummy smiles was reported to be the hyperfunction of the muscles of the upper lip. Previous reports showed that botulinum toxin (Botox) is effective in the treatment of gummy smiles with a reversible effect, rapid initial action, safe application, low risk, and satisfactory outcome. The effect of Botox is usually observed between one and two weeks. This study aims to review the recent updates and guidelines for gummy smile treatment using botulinum toxin. A literature review was conducted involving relevant studies discussing gummy smile treatment using botulinum toxin with no time restriction. The PubMed and Google Scholar databases were used to gather the most relevant studies. The initial screening revealed 62 studies, and after removing the out-of-scope studies, the final review included 28 studies. Botulinum toxin can be used effectively for the treatment of gummy smile caused by lip dynamics with rarely reported complications. However, the most observed limitation was the temporary duration, which was reported to range from four to six months, and the re-injection of botulinum toxin is usually needed.

## Introduction and background

The smile is the most recognized facial expression that contributes to a patient's social skills, self-esteem, and ability to interact with others [1-3]. Excessive gingival exposure (gummy smile) is a non-aesthetic condition characterized by excessive exposure of the gingiva during smiling [1]. Patients' request to treat high smile line has been increasing in the last few years [4]. This includes gingival overexposure, which is called excessive gingival display or gummy smile [4-6]. A gummy smile is a common variation among the population especially in females and has a prevalence of 10.5%-29% [5]. Numerous techniques have been reported to treat gummy smiles; these techniques are mainly gingivectomy, orthognathic surgery, lip repositioning, and botulinum toxin A injections (Botox) [4,7-9]. The treatment of choice depends on etiological factors such as skeletal disorders, short anterior crowns, and muscular hyperactivity [3,4]. The most common cause of gummy smiles was reported to be the hyperfunction of the muscles of the upper lip [10,11]. Gummy smiles due to lip dynamics have been successfully treated with the use of Botox, which reduces the upward movement of the lip [12,13]. Changing lip position and movement with Botox is considered a suitable choice due to the low morbidity and ease of use [12]. Previous studies discussed the treatment of gummy smiles using botulinum toxin; however, most of these studies had a different approach for the treatment depending on the site of injection and dose adjustments. This study aims to review the recent updates and guidelines for gummy smile treatment using botulinum toxin.

## Review

Methods

This article involved a review of relevant published papers discussing the effect of Botox injection on the treatment of gummy smile. Several databases including PubMed and Google Scholar were used to gather the most relevant articles. A search set was applied to combine a range of keywords: botulinum toxin, Botox, and gummy smile. By using this method, all the articles discussing the effect of Botox injection on the treatment of gummy smile were obtained. In the inclusion criteria, we included all the relevant studies discussing gummy smile and their treatment using botulinum toxin. The papers that had a poor methodology and insufficient data were excluded. The initial screening revealed 62 papers. After applying our inclusion criteria, the most relevant articles were selected and used in our current review. This study was conducted by reviewing 28 papers related to the effect of Botox injection on the treatment of gummy smile.

Excessive gingival display (gummy smile)

**Figure 1:**
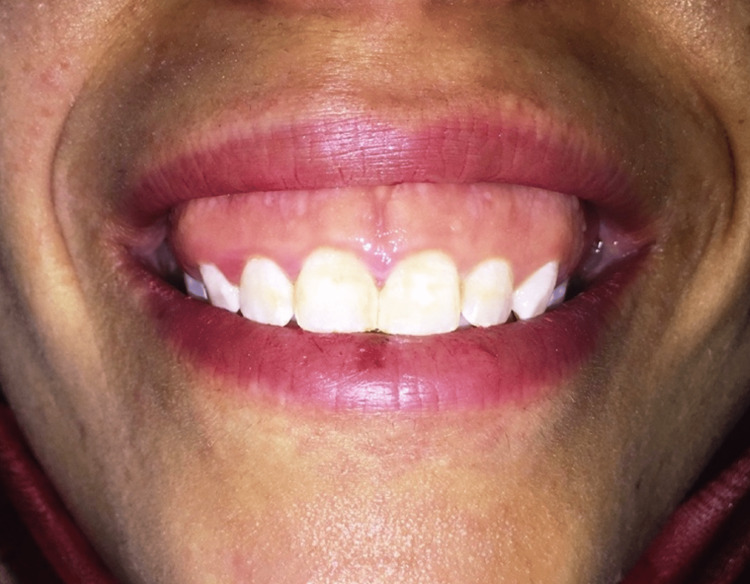
Demonstration of a severe gummy smile while smiling. Source: Open-access article under the Attribution-NonCommercial-NoDerivatives International (CC BY-NC-ND) license (http://creativecommons.org/licenses/by-nc-nd/4.0/) [5]

**Figure 2:**
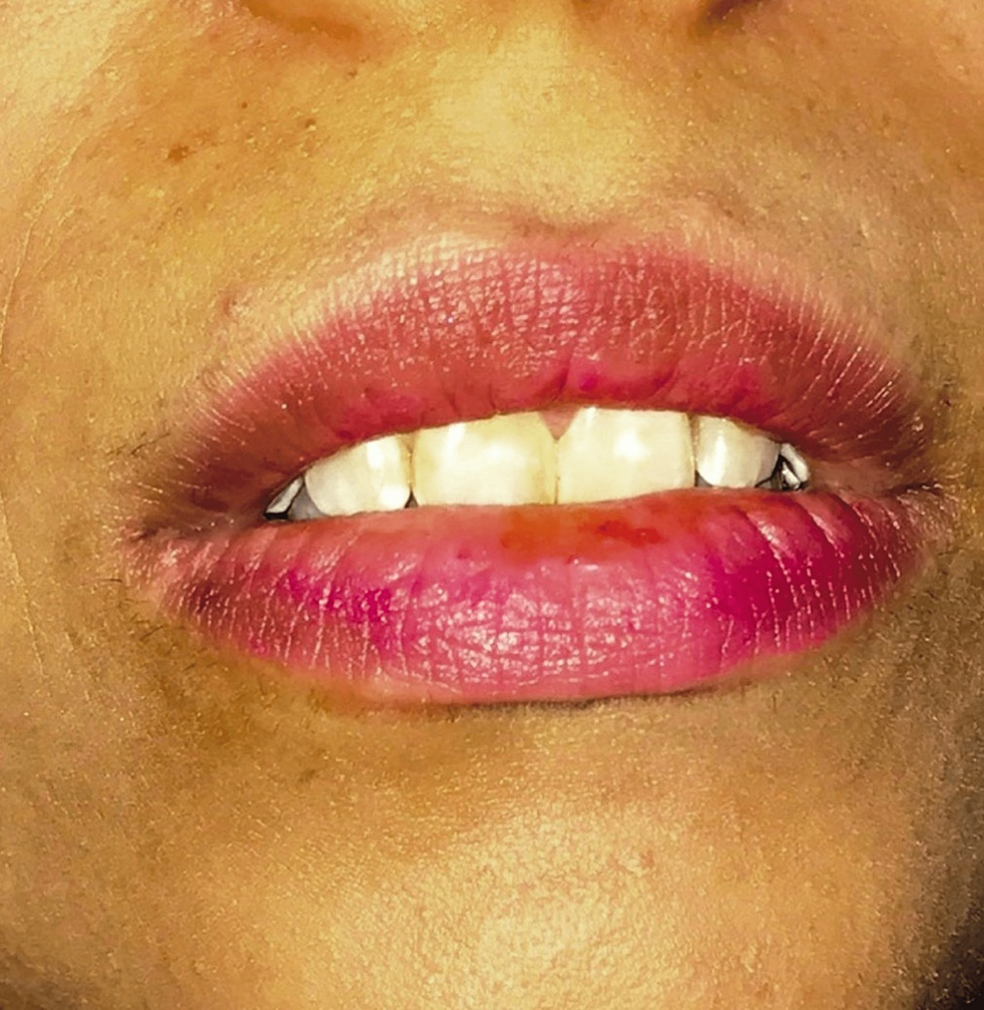
Demonstration of incisors at rest. Source: Open-access article under the Attribution-NonCommercial-NoDerivatives International (CC BY-NC-ND) license (http://creativecommons.org/licenses/by-nc-nd/4.0/) [5]

Gingival display in a certain measure is considered aesthetically acceptable. However, more than 2-3 mm of gingival exposure during smiling is considered to be a gummy smile [3,5,7,14]. The excessive gingival display can be due to altered eruption of the teeth, hyperactive or short upper lip muscles, vertical maxillary excess, or dentoalveolar extrusion [5]. The incidence of gummy smile during smiling is approximately 14% in females and 7% in males [8]. Rubin divided the smile into three types: the full denture smile that is related to the lower depressors and upper retractor muscles causing exposure of all teeth, the Mona Lisa smile that is related to the zygomaticus major muscle, and the canine smile that is associated with an elevation of the upper lip by the action of the levator labii superioris [8]. A classification by Mazzuco and Hexsel categorizes excessive gingival display based on their area [9]. Four types of gummy smiles were presented: posterior, anterior, asymmetric, and mixed gummy smiles [4,8,15]. A posterior gummy smile is associated with normal exposure in the anterior region (<3 mm) and more than 3 mm of exposed gingiva posterior to the canines. An anterior gummy smile is associated with more than 3 mm of exposed gingiva between the canine teeth due to the levator labii superioris alaeque nasi muscles. An asymmetric gummy smile is associated with severe exposure of the gingiva on one site due to asymmetric contraction of either zygomatic or levator labii superioris alaeque nasi muscles. A mixed gummy smile is associated with severe exposure of the gingiva in both the posterior and anterior regions due to a combination of zygomatic and levator labii superioris alaeque nasi muscle movement [4]. The diagnosis of a gummy smile clinically involves the measurement of the width of keratinized gingiva, frenal attachment, clinical crown, vertical limits of the smile, overbite and overjet, anatomic crown length, and probing depth [1,5-7,14]. Skeletal, gingival, and muscular factors should be evaluated while treating gummy smile patients [3]. Radiographic examination is used to locate excessive vertical maxilla, protrusion of maxilla, and bone level [5]. The demonstration of the gingival display while smiling and at rest position is presented in Figures 1-2 [5].

Botulinum toxic A (Botox)

Botulinum toxin was first used in the 1970s for the treatment of excessive muscle contraction and pain. This toxin is typically produced by the bacteria *Clostridium botulinum* [6,15]. *Clostridium botulinum* is an anaerobic and gram-positive rod bacterium with eight serotypes and provides seven botulinum neurotoxins (A-G) [3,4]. Botulinum toxin A (Botox) is mainly used in aesthetic and cosmetic practices. Three types of botulinum toxin A are present, which are called abobotulinum toxin A, incobotulinum toxin A, and onabotulinum toxin A. These are approved by the US Food and Drug Administration. Each of these has a different potency [4]. Botulinum toxin is a minimally invasive approach for the treatment of gummy smiles especially due to hyperactive lip muscles [5,16,17]. The effectiveness of botulinum toxin depends on zinc as each Botox molecule must be associated with a molecule of zinc in order to be effective [15]. Botulinum toxin causes chemical denervation of muscles in the skeletal region, thus resulting in a transitory reduction of muscle activity without systemic manifestations. The injection of botulinum toxin intramuscularly splits the synaptosome-associated protein resulting in stopping the release of acetylcholine (a neurotransmitter that activates gland secretion and muscle contraction) and permitting postsynaptic repolarization. This in turn yields partial chemical denervation. The outcome is a relaxation of the elevating action of the lip and a localized decrease in the activity of the elevator muscle [5,7,8,14,15,18]. Botox was confirmed by previous studies to be a suitable diagnostic method, as well as palliative treatment, for the treatment of gummy smiles [13]. Previous reports showed that Botox is effective in treating gummy smiles with a reversible effect, rapid initial action, safe application, low risk, and straightforward and satisfactory outcome [7,9,17,19,20].

Indication and contraindication

In dentistry and maxillofacial surgery field, botulinum toxin has several indications including improving excessive gingival display and facial wrinkles, tic and tremor treatment, and the management of pain [11,18]. One of the common indications of Botox treatment in gummy smile patients is to reduce the upward movement of the lip [12,13]. Changing lip position and movement with Botox is considered a suitable choice due to the low morbidity and ease of use [12]. Contraindications of using Botox include patients taking calcium channel blockers, pregnant females or those who are lactating, patients taking aminoglycosides or cyclosporine, neuromuscular patients, and patients with hypersensitivity to saline or Botox [5]. The main reported limitation is the duration of these drugs, which lasts approximately four to six months [12]. In case the patient requires surgical intervention, Botox injection is not recommended since botulinum toxin only offers a temporary result for the patient [6].

Treatment planning and patient selection

The main components of assessing gummy smiles are in the full smile and at rest positions. A full smile is divided into two phases, spontaneous and posed phases. Vertical maxillary excess with an excess gingival display at rest is classified into three degrees: Degree 1 is considered 2-4 mm of gingival exposure and can be treated with orthodontic, prosthodontic, and periodontal treatments [12], 4-8 mm is considered as degree 2, and degree 3 is associated with more than 8 mm [12]. Identifying the target muscle associated with a gummy smile is essential to determine the exact treatment choice [4]. Gummy smile can be corrected using Botox. However, the examination of the muscles and the type of smile are essential for accurate treatment planning. The type of Botox used, specific region or muscle, training, technical accuracy, and dosage should be considered and evaluated during the treatment plan phase [7].

Different treatment approach

Patients often lack knowledge and awareness regarding Botox applications in gummy smile; thus, the clinician should discuss all treatment options and their outcomes with the patient [21]. To determine the most favorable treatment approach, previous studies have suggested the classification of gummy smiles based on their etiopathogenic factors. According to these studies, a high smile can be either dentoalveolar type due to excessive vertical or sagittal growth of the upper jaw, dentogingival type that results from a change in the normal path of dental eruption that leads to a reduction in the clinical crown, muscular type due to hyperactivity of the muscles surrounding the oral cavity, or mixed type that results from several causal factors. Moreover, these studies reported that a series of photometric and clinical factors should be considered from the frontal and lateral view during a patient smiling [3].

The height of the central incisors usually ranges from 9.5 to 11 mm based on the patient's gender. In case the patient exhibited excessive gingiva due to short incisors, a referral to a periodontist is usually the next step in order to treat his smile. Thus, it is unacceptable to treat a patient with Botox injections if they suffer from excessive gingival display due to crown exposure [22]. Structural, dento-gingivo-labial, and occlusal parameters should be also evaluated during the treatment plan phase. Structural parameters include the cutting incisal edge of the incisors and the space between the subnasal point. Dento-gingivo-labial parameters include the interlabial gap, the length of the upper lip, and the length of the coronal crown. Occlusal parameters include the inclination of the occlusal plane, overjet, and overbite [3]. Botox can be considered palliative in case surgical treatment is needed, adjuvant treatment in case there is a need for additional treatments such as orthodontic devices or lip augmentation, or remedial in case the cause of excessive gingival display is of muscular origin [9].

Dose adjustment

Dosing can be adjusted depending on the degree of correction desired [12]. The dosage of Botox injection differs from male to female based on the volume of the lip muscle. Typically, males require high units of Botox compared to females to achieve the same effect; this is due to larger muscle volume [5]. Duruel et al. recommended that the dose of botulinum toxin A per region depends on the severity of the gummy smile [4]. Initially, a maximum of 5 IU of onabotulinum toxin A can be injected and was stated to be a safe approach by the authors [4]. In correlation, Gong et al. suggested that the effect of the average dose of Botox for the treatment of gummy smile depends on the patient's gender and the severity of the gummy smile rather than the etiology [23]. Polo investigated botulinum toxin injections with 2.5 units in the levator labii superioris and the levator labii superioris alaeque nasi to treat the neuromuscular excessive gingival display [24]. The author reported that it was satisfactory and effective; however, the effect was temporary with a 5.2 mm mean gingival reduction [24].

Procedure

The treatment of gummy smile using Botox can be divided into three types [7]. The corrective type is performed when the gummy smile is caused by muscle activity. The adjuvant type is performed when a combination of various causes are present and supplementary treatments are necessary such as orthodontic treatment or lip augmentation. The palliative type is indicated whenever surgery is needed [7]. Numerous studies have suggested that botulinum toxin injection in the elevator muscles of the upper lip is an effective treatment approach for gummy smile [4,5,8,19]. A previous study conducted by Cengiz et al. illustrated that Botox injection might be effective in patients with increased gingival exposure [6]. In addition, the levator labii superioris alaeque nasi and the orbicularis oris can be selected for Botox injections [6]. However, Cengiz et al. concluded that both groups evaluated showed a relapse potential when the fourth month was reached [6]. A study by Makkeiah et al. aimed to assess the effectiveness of surgical lip repositioning and botulinum toxin A for the treatment of gummy smile caused by hyperactive lip based on patient satisfaction and the smile outcome [2]. The results showed that botulinum toxin A injection demonstrates better results compared to surgery; in addition, Botox has achieved the desired aesthetic requirement with more satisfactory and safer results compared to surgical lip repositioning [2].

The Yonsei point is considered an effective point for intramuscular Botox [1,5,25]. This point is positioned in the middle of a triangle shaped by the zygomaticus minor, levator labii superioris alaeque nasi, and levator labii superioris muscles [5]. Previous studies suggested that the injection of botulinum toxin A at the Yonsei point could be a predictable and reliable treatment choice for different types of gummy smiles [4,25]. Moreover, Suber et al. reported that the levator labii superioris alaeque nasi and levator labii superioris muscles are considered effective sites for treatment using Botox [8]. A recent clinical trial by Costa et al. aimed to assess the effects of Botox treatment on gummy smile reduction, patient satisfaction, and muscle activity [26]. The results showed that increasing the number of injection points will result in patient satisfaction, gingival display reduction, and a persistent Botox effect. However, the overall intensity of the outcome did not increase [26]. In addition, Aldhaher and Bede recommended the use of a four-point technique for a better outcome regarding the degree of satisfaction and clinical measurements compared to a two-point technique [10]. Myotomy with lip repositioning was reported to provide more stability in the long term compared to repositioning without myotomy. Yet, few patients considered the result to be satisfying [15]. Relevant clinical studies discussing the use of Botox for gummy smile treatment are presented in Table 1.

**Table 1:** Relevant clinical trials discussing the use of Botox for gummy smile treatment.

Authors	Year of publication	Dose	Number of patients	Technique	Outcome
Duruel et al.	2019	5 IU	3	Injection at Yonsei points	The dose of botulinum toxin (BT) A per region depends on the severity of the gummy smile. Initially, a maximum of 5 IU onabotulinum toxin A can be injected and was stated to be a safe approach by the author
Cengiz et al.	2020	5 IU	28	Injection at the levator labii superioris alaeque nasi and orbicularis oris muscles	Botox injection might be effective in patients with increased gingival exposure. In addition, levator labii superioris alaeque nasi and orbicularis oris can be selected for Botox injections. However, both groups evaluated showed a relapse potential when the fourth month was reached
Suber et al.	2014	5 IU	14	Injection of BT into the lip elevator muscles	The levator labii superioris alaeque nasi and levator labii superioris muscles are considered effective sites for treatment using Botox
Mazzuco and Hexsel	2010	2.5 and 5 IU	16	Injection of BT based on the main muscles involved	The overall average improvement of gummy smile was 75.09%
Aldhaher and Bede	2022	2.5 and 5 IU	40	Two groups: the first group received 2.5 IU injection at one point per side and the second group received 5 IU of BT at two points per side	The second group showed a better result in terms of the degree of satisfaction and clinical measurements
Shemais et al.	2021	3 IU	25	BT injection at Yonsei points with and without zinc supplementation prior to injections	Zinc supplement prior to BT injection can enhance clinical efficacy and maintain its effect
Skaria et al.	2020	2.5 IU	20	Injected on either side of the face	Gingival exposure reduction from 4.93 to 3.705 mm with a decrease in the nasolabial fold
Gong et al.	2021	2 IU	29	Bilateral single-point injections	The effect of the average dose of Botox for the treatment of gummy smile depends on the patient's gender and the severity of the gummy smile rather than the etiology
Polo	2008	2.5 IU	30	Injection at Yonsei points	BT A injection is statistically superior and effective to baseline smiles; however, it was transitory

**Figure 3:**
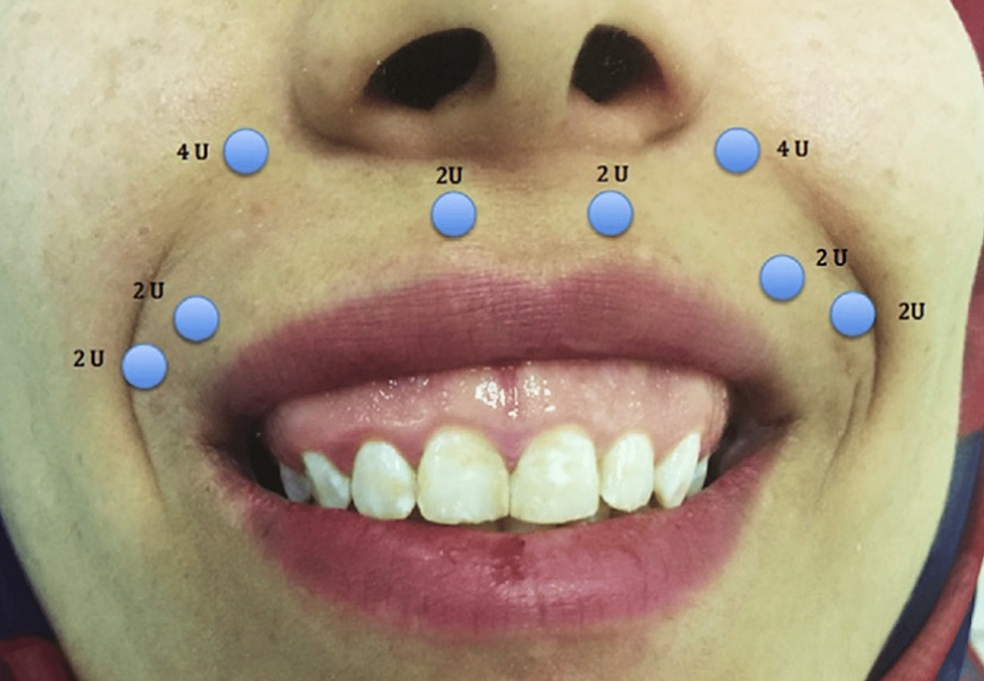
Injection sites of 20 units of Botox. Source: Open-access article under the Attribution-NonCommercial-NoDerivatives International (CC BY-NC-ND) license (http://creativecommons.org/licenses/by-nc-nd/4.0/) [5]

The anterior gummy smile can be injected on the lateral side to the wing of the nose. Posterior gummy smile injection should also be applied laterally to the wing of the nose; however, half of the dose should be used, and the second point should be injected laterally by 2 cm to the first, particularly at the tragus line level [18]. A mixed gummy smile injection is done by using both previous techniques. However, a reduction of the dose by 50% on the lateral side to the wing of the nose is recommended [18]. Injection sites are presented in Figure 3 [5].

Findings

**Figure 4:**
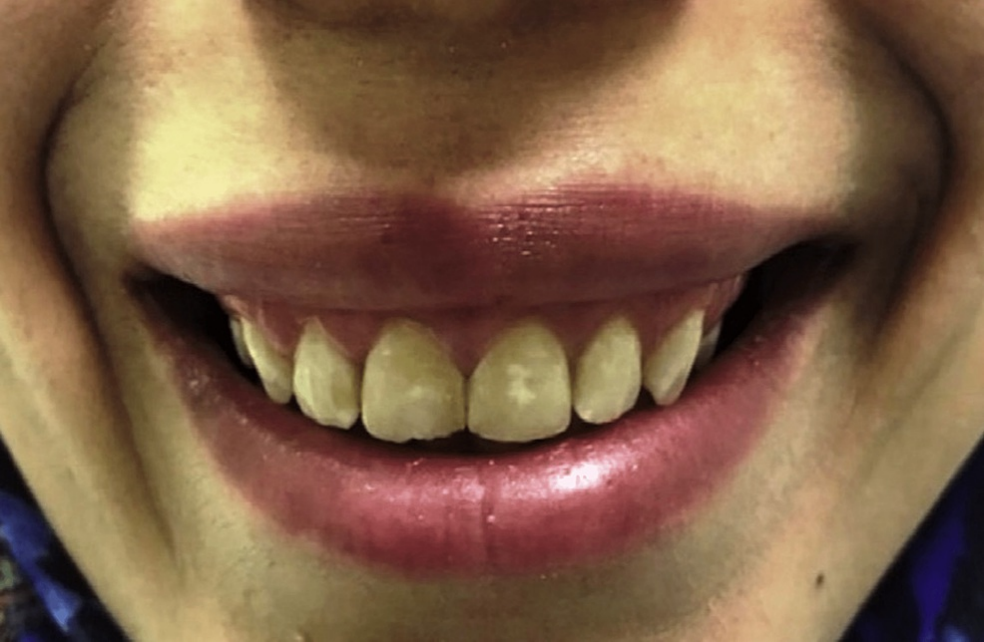
Two weeks following Botox injections. Source: Open-access article under the Attribution-NonCommercial-NoDerivatives International (CC BY-NC-ND) license (http://creativecommons.org/licenses/by-nc-nd/4.0/) [5]

A previous systematic review by Zengiski et al. aimed to evaluate the longevity and effectiveness of the botulinum toxin for the treatment of excessive gingival display [27]. The author reported that Botox is considered an alternative treatment approach for reducing gummy smiles mainly in cases that the gummy smile was up to 4 mm [27]. The effect of Botox is usually observed between one and two weeks and lasts for four to six months [5,11]. A systematic review by Chagas et al. reported a significant effect of Botox on the treatment of gummy smiles [7]. In addition, the stability was constant until the eighth week [7]. However, the gingival display may reoccur after 12 weeks [7]. Moreover, Razmaite et al. reported up to 5 mm reduction in gummy smile following Botox injections [1]. The author reported that the best outcome was observed after two weeks of injection and the effect remains for approximately three months or more [1]. Oliveira et al. also reported a decrease in the surface electromyography signal amplitude after two to four weeks post-Botox injections [28]. Follow-up after two weeks following Botox injections is presented in Figure 4 [5].

Complications, side effects, and recommendations

Complications associated with Botox are reported to be uncommon and most likely temporary. Most of the guidelines recommend 2.0-2.5 units to be injected in a particular muscular region [12]. Botox re-injections should be avoided in case the clearance of the previous effect has not worn off completely to prevent antibody formation that can result in unsatisfactory results. Zengiski et al. stated that after 24 weeks of injection, the values return to their initial [27]. Botox injections are generally considered safe in cases that quantity and technique are measured correctly. Yet, limited localized side effects were reported previously including inflammation, nerve palsy, pain, hematoma, infection, bruising, edema, and loss of muscle strength. Moreover, the inappropriate technique of injection could lead to speech difficulties, asymmetric unpleasant smile appearance, and drinking or chewing problems. Overdose might result in ptosis or drooping of the lip leading to coverage of the teeth on smiling [5].

## Conclusions

Botulinum toxin is considered a minimally invasive approach for the treatment of gummy smiles particularly due to hyperactive lip muscles. This review demonstrates that the botulinum toxin can be used effectively for the treatment of gummy smile caused by lip dynamics with rarely reported complications. In addition, the effect is mainly between one and two weeks with great patient satisfaction. However, the most common limitation of this procedure is the temporary duration of Botox, which ranges from four to six months; thus, re-injection of botulinum toxin is usually needed. Moreover, the clinician has the responsibility to provide a satisfactory treatment outcome while also explaining all the potential side effects and the limitation of this procedure.
